# Treatment response in chronic urticaria: analysis of clinical and laboratory predictors^؆^

**DOI:** 10.1016/j.abd.2025.501261

**Published:** 2026-01-23

**Authors:** Joice Trigo da Fonseca, Joanemile Pacheco de Figueiredo, Leila Vieira Borges Trancoso Neves, José Carlisson Santos de Oliveira, Janinne Souza de Oliveira, Vitória Rani Figueiredo, Régis de Albuquerque Campos

**Affiliations:** Centro de Referência em Urticária, Complexo Hospitalar Universitário Professor Edgard Santos, Universidade Federal da Bahia, Salvador, BA, Brazil

**Keywords:** Biomarkers, Chronic urticaria, Histamine antagonists, Omalizumab

## Abstract

**Background:**

Chronic urticaria (CU) compromises quality of life, requiring escalated treatment with second-generation H1 antihistamines, omalizumab, and, in refractory cases, cyclosporine. Predictors of therapeutic response are not yet well established.

**Objective:**

To evaluate clinical and laboratory factors associated with treatment response in patients with CU.

**Methods:**

Cross-sectional study of 175 patients with CU followed at the Urticaria Reference Center (UCARE) of Complexo Hospitalar Universitário Professor Edgard Santos (HUPES/UFBA) between 2023 and 2024. Sociodemographic, clinical, and laboratory data were analyzed. Treatment response was assessed using the Urticaria Control Test (UCT), with responders being those with a score ≥12 or Angioedema Control Test (AECT) ≥10.

**Results:**

Most patients were female (80.6%), with a mean age of 45.3 years. Chronic spontaneous urticaria (CSU) was predominant (86.3%). Higher body mass index (BMI), early onset, longer disease duration, and psychiatric disorders were associated with poorer response to second-generation H1 antihistamines. Responders to these drugs had shorter disease duration and a lower proportion of women compared to those requiring omalizumab. In omalizumab users, mental disorders remained associated with refractoriness. Total IgE, eosinophils, CRP, ESR, D-dimer, and anti-TPO did not correlate with therapeutic response.

**Study limitations:**

Cross-sectional study and reliance on clinical records, information bias, and selection bias.

**Conclusions:**

High BMI, female gender, early symptom onset, prolonged disease duration, and mental disorders were associated with poorer response to CU treatment. The evaluated laboratory tests did not demonstrate predictive value for treatment response.

## Introduction

Chronic urticaria (CU) is characterized by erythematous and pruritic plaques, accompanied or not by angioedema, lasting more than six weeks. It can be classified as chronic spontaneous urticaria (CSU) when it occurs without a specific trigger, or chronic inducible urticaria (CIndU) when it occurs after a specific stimulus, primarily physical. Its course and duration are unpredictable, ranging on average from six months to five years.^1^ It is estimated that CSU affects 0.5% to 1% of the global population, which is equivalent to two-thirds of CU cases, negatively impacting patients' quality of life.^2^

The pathophysiology of CU has not yet been fully elucidated, but the role of histamine, as well as some pro-inflammatory cytokines released by mast cells and basophils, is indisputable. The main mechanism associated with CSU is autoimmune, based on the presence of class G autoantibodies directed at high-affinity IgE receptors (FceRI) on mast cells and basophils or at circulating IgE molecules, in addition to IgE autoantibodies directed at various endogenous antigens.^3^ The pathogenesis of CIndU is not yet fully understood, but the mechanisms involved in CSU also seem to play an important role.^4^

Therapeutic management is escalated according to severity and refractoriness to treatment and includes the use of second-generation H1 antihistamines at standard doses as recommended in the package insert up to quadruple doses, anti-IgE monoclonal antibody (omalizumab), and cyclosporine.^1^

Despite advances in treatment, CU management is still based on a trial-and-error approach, with little ability to predict which patients will respond to a given treatment phase, which contributes to a longer time to achieve symptom control and increases the costs associated with therapy.^5^

In recent years, several studies have sought to identify clinical and laboratory markers capable of predicting therapeutic response in patients with chronic urticaria, particularly regarding the second-generation H1 antihistamines, omalizumab, and cyclosporine. According to a systematic review by Fok et al.,^5^ elevated Urticaria Activity Score (UAS7) values, as well as increased serum CRP and D-dimer levels, are associated with a lower response to second-generation H1 antihistamines. Regarding omalizumab, low IgE levels, a positive Autologous Serum Test (AST), and a positive Basophil Histamine Release Test (BHRA) were identified as possible predictors of poor or delayed response. In contrast, in the case of cyclosporine, there is evidence that a positive BHRA and reduced IgE levels may indicate a better response to treatment.

Complementarily, Giménez-Arnau et al.^6^ also observed that high disease activity, increased CRP, ESR, and D-dimer levels are the main predictors of an absent or unsatisfactory response to second-generation H1 antihistamines. Furthermore, low or very low baseline IgE, a positive AST, a positive basophil activation/histamine release test (BAT/BHRA+), basopenia, eosinopenia, and elevated D-dimer were associated with an absence of response to omalizumab but a good response to cyclosporine. Conversely, normal or slightly elevated baseline IgE levels, as well as increased expression of the FcεRI receptor on basophils, seem to indicate a more rapid response to omalizumab.

Despite these findings, there is still little solid evidence in the literature on clinical and laboratory markers that can practically guide therapeutic choices, especially in specific contexts such as in Brazil. Therefore, further studies to validate these findings in different populations are needed.

Therefore, the present study aims to analyze possible clinical and laboratory predictors of treatment response in patients with chronic urticaria treated at a referral center, aiming to contribute to more personalized and efficient disease management.

## Methods

### Study design

This is a cross-sectional, observational study conducted with patients diagnosed with Chronic Urticaria (CU) and treated at the Urticaria Reference Center (UCARE), linked to Complexo Hospitalar Universitário Professor Edgard Santos (HUPES) of Universidade Federal da Bahia (UFBA), in Salvador, Bahia, between January 2023 and November 2024.

Patients of both sexes, without age restrictions, with a clinical diagnosis of CU, defined as the presence of wheals and/or angioedema for more than six weeks, according to the international guidelines of EAACI/GA²LEN/EDF/WAO, were included.^1^ In cases of CIndU, specific provocation tests were performed using standardized instruments such as the FricTest® and TempTest®.^7^

The sample was selected by convenience, and patient records were reviewed according to a standardized protocol to collect information on demographic data, clinical characteristics, laboratory results, administered treatments, and specificities of CU. No prior sample size calculation was performed, as the objective was descriptive and exploratory, with an analysis of all cases treated at the referral center during the proposed timeframe.

Patients with other urticarial lesions that did not correspond to CU, lack of follow-up, or those who did not consent to participate in the study were excluded. Of the patients approached, only two declined to participate.

### Data collection and laboratory tests

Data collected included sex, age, BMI, diagnosis (CSU, CIndU, or CSU + CIndU), disease duration, age at symptom onset, presence of angioedema, hypersensitivity to nonsteroidal anti-inflammatory drugs (NSAIDs), associated comorbidities (metabolic, autoimmune, psychiatric, atopic, cardiovascular, and mental health), and laboratory tests.

The following tests were evaluated: total IgE (values ​​< 40 IU/mL considered low),^5^ eosinophils (<50 cells/μL defined as eosinopenia),^8^ CRP, ESR, D-dimer, and anti-TPO. Tests were performed at the Immunology and Molecular Biology Laboratory of the Institute of Health Sciences at UFBA or at other services affiliated with the Brazilian Unified Health System (SUS, Sistema Único de Saúde) or private laboratories. In patients receiving omalizumab, only IgE values ​​obtained before the first dose were considered.^9^

### Response monitoring

Therapeutic response was assessed using the Urticaria Control Test (UCT), with a score ≥ 12 considered adequate control.^10^ In cases of isolated angioedema, the Angioedema Control Test (AECT) was used, with a score ≥10 considered satisfactory.^11^ The UCT was chosen because it is easier to apply and understand by patients during consultations and is administered under medical supervision at the time of care. Although the UAS7 was initially considered, its administration requires daily completion by the patient for seven consecutive days before the consultation, which proved difficult to adhere to and follow, and could compromise data reliability.

### Treatment aspects

Patients were categorized into four treatment phases, according to current management: Phase 1, with second-generation H1 antihistamines at standard or double doses; Phase 2, with second-generation H1 antihistamines at triple or quadruple doses; Phase 3, with omalizumab; and Phase 4, with immunosuppressants. The minimum length of duration of each Phase (1 and 2) was four weeks, with variations depending on appointment availability and patient flow.

For the response to omalizumab, patients who achieved a UCT ≥ 12 were considered responders; partial responders were those who did not achieve this score but showed an increase of ≥ 3 points in the UCT (minimum clinically significant difference);^12^ and non-responders were those who did not demonstrate improvement in the score after at least six months of continuous use of the medication.

Patients responding to omalizumab were further classified according to the time to response: early response, when improvement was observed after the first application; and late response, when improvement occurred only after subsequent applications.

### Statistical analysis

Statistical analysis was performed using the chi-square test, Fisher's exact test, Student's *t*-test, Mann-Whitney test, or Kruskal-Wallis test, depending on the nature and distribution of the variables. ROC curves and effect size measures, such as Cramer's V, Cohen's d, and chi-square, were used, following recommendations in the literature. All analyses were conducted using R software version 4.3.3 (R Core Team, 2023) and considered a significance level (α) of 5%.

### Ethical aspects

The study was approved by the Research Ethics Committee of Complexo Hospitalar Universitário Professor Edgard Santos (CAAE: 65818222.9.0000.0049). Patients were invited to participate during their scheduled appointments with their attending physician, at which time they were presented with the Informed Consent Form (ICF), authorizing both the collection of additional data and review of medical records.

## Results

### Sample characterization

A total of 175 patients with chronic urticaria were included, 80.6% of whom were female, with a mean age of 45.3 years. CSU was the most common subtype (55.4%), followed by a combination of CSU and CIndU (30.9%), and CIndU alone in 13.7%. Angioedema was present in 80% of cases, with the most common clinical presentation of CSU being a combination of urticaria and angioedema (73%; Table 1).

**Table 1:** Demographic and clinical data.

Variable	Results
**Female sex, n (%)**	141 (80.5)
**Age in years (n = 175)**	
Mean (SD)	45.2 (18.02)
Median (Q1; Q3)	47.14 (37.00; 59.50)
Range	6 – 85.77
BMI (KG/ BMI (kg/m²), (n = 175*):	
Mean (SD)	27.92 (6.32)
Median (Q1; Q3)	27.60 (23.75; 32.05)
Range	13.30 ‒ 47
**BMI category (n = 175), n (%)**	
Overweight	60 (34.29)
Obesity	58 (33.14)
Normal weight	50 (28.57)
Underweight	7 (4.00)
Age at onset in years (n = 171*):	
Mean (SD)	32.80 (18.08)
Median (Q1; Q3)	33.00 (19.00; 48.00)
Range	0 ‒ 75
**Disease duration in years:**	
Mean (SD)	11.92 (12.60)
Median (Q1; Q3)	8.00 (3.00; 16.50)
Range	0‒77
**Angioedema (n = 175) – n (%)**	141 (80.57)
**Main diagnosis (n = 175) – n (%)**	
CSU	97 (55.43)
CSU + CindU	54 (30.86)
CindU alone	24 (13.71)
**Clinical manifestation (n = 175) – n (%)**	
Urticaria alone	34 (19.42)
Urticaria + angioedema	129 (73.71)
Angioedema alone	12 (6.85)
**CIndU specification (n = 78) – n (%)**	
Dermographism	54 (69.23)
Pressure	17 (21.79)
Heat	11 (14.10)
Solar	4 (5.13)
Cholinergic	4 (5.13)
Cold	1 (1.28)
**Comorbidities (n = 175) – n (%)**	
Metabolic disorder	83 (47.43)
Atopic disorder	70 (40.00)
Cardiovascular disorder	57 (32.57)
Hypersensitivity to NSAIDs	55 (31.43)
Autoimmune disorder	40 (22.86)
Mental disorder	32 (18.29)

SD, Standard Deviation; Q1, First Quartile (25th percentile); Q3, Third Quartile (75th percentile); BMI, Body Mass Index; CSU, Chronic Spontaneous Urticaria; CIndU, Chronic Inducible Urticaria; NSAIDs, Nonsteroidal Anti-inflammatory Drugs.

The most prevalent comorbidities were: metabolic disorders (47.4%), atopic disorders (40%), cardiovascular disorders (33%), NSAID hypersensitivity (31%), autoimmune disorders (23%), and mental disorders (18%; Table 1).

**Fig. 1:**
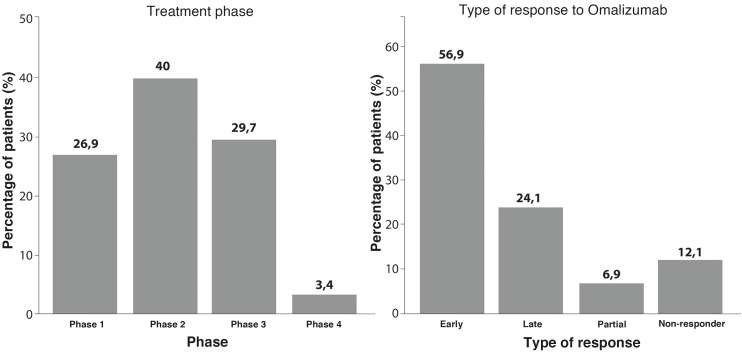
Treatment data.

Regarding treatment, 66.9% were exclusively using second-generation H1 antihistamines, 29.7% were using omalizumab, and 3.4% were using immunosuppressants. The distribution by phase was: 26.9% in Phase 1, 40% in Phase 2, 29% in Phase 3, and 3.4% in Phase 4. Only approximately 10% of patients responded to the standard dose of second-generation H1 antihistamine. Among those who used omalizumab, 56.9% had an early response, 24% had a late response, 12% did not respond, and 6.8% had a partial response (Fig. 1).

Laboratory tests revealed abnormalities in part of the sample: elevated CRP in 21.9%, ESR in 47.3%, D-dimer in 24.2%, and anti-TPO in 19.6%. The mean eosinophil count was 196.8 cells/μL, with eosinopenia in 17% of cases. Total IgE averaged 272 IU/mL, with 73.8% of patients showing levels ≥ 40 IU/mL (Table 2).

**Table 2:** Laboratory data.

Variable	Results
**High ESR (n = 150) – n (%)**	71 (47.33)
**Positive CRP (n = 160) – n (%)**	35 (21.88)
**High** D-**Dimer (n = 62) – n (%)**	15 (24.19)
**Eosinophils (n = 164):**	
Mean (SD)	196.83 (189.96)
Median (Q1; Q3)	138.50 (68.96; 273.25)
Range	0–1.112
≥50 – n (%)	135 (82.31)
<50 – n (%)	29 (17.68)
**Total IgE (n = 130):**	
Mean (DP)	272 (448.4)
Median (Q1, Q3)	108.5 (39.1; 291.0)
Range	2–2.500
≥40 – n (%)	96 (73.8)
<40 – n (%)	34 (26.2)
**High Anti-TPO (n = 153) – n (%)**	30 (19.61)

SD, Standard Deviation; Q1, First Quartile (25th percentile); Q3, Third Quartile (75th percentile); ESR, Erythrocyte Sedimentation Rate; CRP, C-Reactive Protein; TPO, Thyroperoxidase.

### Comparative analyses

The comparative analysis between patients who responded to the standard or doubled dose of second-generation H1 antihistamines and those who required increased doses revealed no statistically significant differences regarding demographic and clinical variables, presence of comorbidities, or laboratory tests evaluated (Table 3).

**Table 3:** Comparison between patients responding to standard or double doses of H1 antihistamines and those requiring higher doses (n = 86).

Variable	Responds to standard or double dose (n = 39)	Responds only to higher doses (n = 47)	p	ES
**Female sex, n (%)**	28 (71.79)	35 (74.47)	0.973^a^	0.004
**Age in years:**				
Mean (SD)	44.37 (20.46)	46.01 (19.47)	0.591^b^	−0.058
**BMI (kg/m^2^):**				
Mean (SD)	25.77 (5.51)	27.62 (6.84)	0.176^c^	−0.295
**Age at symptom onset:**				
Mean (SD)	30.86 (19.46)	37.26 (19.0)	0.151^b^	−0.158
**Time of disease (years):**				
Mean (SD)	12.22 (16.0)	9.02 (11.3)	0.174^b^	0.149
**Presence of angioedema, n (%)**	29 (74.4)	41 (87.2)	0.212^a^	0.135
**Main diagnosis n (%)**			0.959^a^	0.031
CSU	23 (59.0)	29 (61.7)		
CSU + CIndU	11 (28.2)	12 (25.5)		
CIndU alone	5 (12.8)	6 (12.8)		
**Comorbidities n (%):**				
Metabolic disorder	15 (38.5)	24 (51.1)	0.342^a^	0.103
Autoimmune disorder	12 (30.8)	13 (27.7)	0.938^a^	0.008
Mental disorder	4 (10.3)	3 (27.7)	0.907^a^	0.028
Atopic disorder	18 (46.2)	20 (42.6)	0.907^a^	0.013
Cardiovascular disorder	14 (35.9)	11 (23.4)	0.302^a^	0.111
Hypersensitivity to NSAIDs	13 (33.3)	14 (29.8)	0.905^a^	0.013
**ESR – n (%):**			0.433^a^	0.094
High	12 (44.4)	24 (57.1)		
Normal	15 (55.56)	18 (42.86)		
**CRP – n (%):**			0.169^b^	0.049
Positive	7 (19.4)	6 (13.3)		
Normal	29 (80.56)	39 (86.67)		
**D-dimer – n (%):**			1.000^d^	0.000
High	3 (21.4)	4 (28.6)		
Normal	11 (78.57)	10 (71.43)		
**Eosinophils (cells/μL):**				
Mean (SD)	151.4 ± 139.2	210.6 ± 192.4	0.169^b^	−0.152
**Total IgE (UI/mL):**				
Mean (SD)	230.5 (288.17)	332.9 (579.11)	0.834^b^	−0.026
< 40 UI/mL – n (%)	8 (29.63**)**	10 (23.81)	0.798^a^	0.031
≥ 40 UI/mL – n (%)	19 (70.4)	32 (76.2)	0.798^a^	0.031
**Anti-TPO – n (%):**			1.000^a^	0.000
High	7 (21.2)	8 (20.0)		
Normal	26 (78.79)	32 (80.00)		

SD, Standard Deviation; BMI, Body Mass Index; CSU, Chronic Spontaneous Urticaria; CIndU, Chronic Inducible Urticaria; NSAIDs, Non-Steroidal Anti-Inflammatory Drugs; ESR, Erythrocyte Sedimentation Rate; CRP, C-Reactive Protein; TPO, Peroxidase.

ES, Effect Size. The following effect sizes were calculated: Cohen's d, for the independent *t*-test; r, for the Mann-Whitney test; Cramer's V, for the Fisher's exact test and chi-square test of independence.

a Chi-Square test of independence.

b Mann-Whitney Test.

c Independent *t*-test.

d Fisher’s Exact Test.

On the other hand, when comparing patients who responded to second-generation H1 antihistamines with those who required omalizumab, significant differences were observed in terms of sex and disease duration. The proportion of women was higher among those receiving omalizumab, while patients who responded to H1 antihistamines had a shorter mean disease duration. The other variables analyzed showed no statistical differences between the groups (Table 4).

**Table 4:** Comparison of the clinical and laboratory profile of patients responding to antihistamines and those responding to Omalizumab (n = 133).

Variable	Responds to antihistamines (n = 86)	Responds to Omalizumab (n = 47)	p	ES
**Sex, n (%):**			**0.009**^a^	0.226
Female	63 (73.26)#	44 (93.62)#		
Male	23 (26.74)#	3 (6.38)#		
**Age in years:**			0.596^b^	0.046
Mean (SD)	45.27 (19. 82)	45.37 (16. 40)		
Median (Q1; Q3)	49.50 (33.22; 60.00)	45.95 (38.37; 57.61)		
**BMI (kg/m^2^):**			0.068^c^	−0.333
Mean (SD)	26.78 (6.31)	28.88 (6.28)		
Median (Q1; Q3)	27.55 (23.00; 30.42	45.95 (38.37; 57.61)		
**Age at Onset in years:**			0.097^b^	0.146
Mean (SD)	34.41 (19.36)	29.15 (15.75)		
Median (Q1; Q3)	40.00 (15.00; 49.50)	30.00 (17.50; 36.75)		
**Time of disease:**			**<0.001**^b^	−0.310
Mean (SD)	10.45 (13.62)	15.11 (11.35)		
Median (Q1; Q3)	7.00 (3.00; 10.00)	11.00 (6.00; 21.00)		
**Angioedema n (%):**	70 (81.40)	41 (87.23)	0.534^a^	0.054
**Main diagnosis n (%):**			0.668^a^	0.078
CSU	52 (60.74)	26 (55.32)		
CSU + CIndU	23 (26.74)	16 (34.04)		
CIndU alone	11 (12.79)	5 (10.64)		
**Comorbidities n (%):**			0.653^a^	0.653^a^
Metabolic disorder	39 (45.35)	24 (51.06)	0.106^a^	0.140
Autoimmune disorder	25 (29.07)	7 (14.89)	1.000^d^	0.000
Mental disorder	7 (8.14)	4 (8.51)	0.232^a^	0.104
Atopic disorder	38 (44.19)	15 (31.91)	0.255^a^	0.099
Cardiovascular disorder	25 (29.07)	19 (40.43)	1.000^a^	0.000
Hypersensitivity to NSAIDs – n (%)	27 (31.40)	14 (29.79)		
**ESR n (%):**			0.460^a^	0.070
High	36 (52.17)	19 (43.18)		
Normal	33 (47.83)	25 (56.82)		
**CRP, n (%):**			0.140^a^	0.132
Positive	13 (16.05)	13 (28.89)		
Normal	68 (83.95)	32 (71.11)		
**D-Dimer, n (%):**			1.000^a^	0.000
High	7 (25.00)	5 (25.00)		
Normal	21 (75.00)	15 (75.00)		
**Eosinophils:**			0.300^b^	−0.093
Mean (SD):	185.36 (173.26)	230.28 (238.30)		
Median (Q1; Q3):	137.00 (61.50; 255.75)	153.45 (92.88; 280.25)		
**Total IgE:**				
Mean (SD)*	292.85 (486.31)	331 (506.5)	0.740^b^	−0.033
Median (Q1; Q3)	111.0 (39.5; 313.0)	111.0 (64.8; 419.5)	0.740^b^	−0.033
< 40 – n (%)	18 (26.09)	6 (18.8)	0.579^a^	0.055
≥ 40 – n (%)	51 (73.91)	26 (81.2)	0.579^a^	0.055
**Anti –TPO, n (%):**			1.000^a^	0.000
High	15 (20.55)	9 (20.00)		
Normal	58 (79.45)	36 (80.00)		

SD, Standard Deviation; Q1, First Quartile (25th percentile); Q3, Third Quartile (75th percentile); BMI, Body Mass Index; CSU, Chronic Spontaneous Urticaria; CIndU, Chronic Inducible Urticaria; NSAIDs, Non-Steroidal Anti-Inflammatory Drugs; ESR, Erythrocyte Sedimentation Rate; CRP, C-Reactive Protein; TPO, Peroxidase.

ES, Effect Size. The following effect sizes were calculated: Cohen's d, for the independent *t*-test; r, for the Mann-Whitney test; Cramer's V, for the Fisher's exact test and chi-square test of independence.

a Chi-Square test of independence.

b Mann-Whitney Test.

c Independent *t*-test.

d Fisher’s Exact Test.

When analyzed stratified by treatment phase, it was observed that among patients who responded to second-generation H1 antihistamines at standard or double doses (Phase 1), BMI was significantly lower compared to non-responders. No other variables showed significant differences (Table 5).

**Table 5:** Comparison of patients who responded versus non-responders to Phase 1 regarding their clinical and laboratory profile (n = 174).

Variable	No (n:135)	Yes (n:39)	p	ES
**Sex – n (%):**			0.187^a^	0.100
Female	112 (82.96)	28 (71.79)		
Male	23 (17.04)	11 (28.21)		
**Age in years:**			0.887^b^	0.011
Mean (SD):	45.53 (17.39)	44.37 (20.46)		
Median (Q1; Q3):	48.00 (38.00; 59.50)	47.00 (33.14; 59.50)		
**BMI (kg/m^2^)**			**0.015**^c^	0.445
Mean(DP):	28.55 (6.44)	25.77 (5.51)		
Median (Q1; Q3):	27.90 (23.85; 32.50)	27.10 (22.60; 29.05)		
**Age of onset in years**			0.528^b^	0.048
Mean (SD):	33.26 (17.76)	30.86 (19.46)		
Median (Q1; Q3):	34.00 (20.00; 48.00)	29.00 (15.00; 47.00		
**Time of disease in years:**			0.711^b^	0.028
Mean (SD):	11.90 (11.58)	12.22 (16.00)		
Median (Q1; Q3):	8.00 (4.00; 17.00)	7.00 (3.00; 13.00)		
**Angioedema – n (%)**	112 (82.96)	29 (74.36)	0.329^a^	0.074
**Main diagnoses– n (%):**			0.899^a^	0.035
CSU	74 (54.81)	23 (58.97)		
CSU + CIndU	42 (31.11)	11 (28.21)		
CIndU alone	19 (14.07)	5 (12.82)		
**Comorbidities, n (%):**				
Metabolic disorder	68 (50.37);	15 (38.46);	0.259^a^	0.086
Autoimmune disorder	28 (20.74);	12 (30.77);	0.273^a^	0.083
Mental disorder	28 (20.74);	4 (10.26);	0.210^a^	0.095
Atopic disorder	51 (37.78);	18 (46.15)	0.450^a^	0.057
Cardiovascular disorder	43 (31.85);	14 (35.90)	0.779^a^	0.021
Hypersensitivity to NSAIDs	42 (31.11)	13 (33.33)	0.946^a^	0.005
**ESR – n (%):**			0.876^a^	0.013
High	59 (48.36)	12 (44.44)		
Normal	63 (51.64)	15 (55.56)		
**CRP – n (%):**			0.864^a^	0.014
Positive	28 (22.58)	7 (19.44)		
Normal	96 (77.42)	29 (80.56)		
**D-dimer – n (%):**			1.000^d^	0.000
High	12 (25.00)	3 (21.43)		
Normal	36 (75.00)	11 (78.57)		
**Eosinophils:**			0.119^b^	0.122
Mean (SD):	210.61 (200.32)	151.41 (139.23)		
Median (Q1; Q3):	144.50 (83.38; 278.00)	119.00 (59.50; 170.00)		
**Total IgE:**				
Mean (SD):	289 (482.2)	230.53 (288.17)	0.841^2^	−0.018
Median (Q1; Q3):	105,0 (40,4; 281,0)	109,0 (36,4; 309,5)	0,841^b^	−0,018
< 40 – n (%)	26 (25,2)	8 (29,63)	0,829^a^	0,019
≥ 40 – n (%)	77 (74,8)	19 (70,37)	0,829^a^	0,019
**Anti –TPO– n (%):**			1,000^a^	0,000
High	23 (19,33)	7 (21,21)		
Normal	96 (80,67)	26 (78,79)		

SD, Standard Deviation; Q1, First Quartile (25th percentile); Q3, Third Quartile (75th percentile); BMI, Body Mass Index; CSU, Chronic Spontaneous Urticaria; CIndU, Chronic Inducible Urticaria; NSAIDs, Non-Steroidal Anti-Inflammatory Drugs; ESR, Erythrocyte Sedimentation Rate; CRP, C-Reactive Protein; TPO, Peroxidase.

ES, Effect Size. The following effect sizes were calculated: Cohen's d, for the independent *t*-test; r, for the Mann-Whitney test; Cramer's V, for the Fisher's exact test and chi-square test of independence.

a Chi-Square test of independence.

b Mann-Whitney Test.

c Independent *t*-test.

d Fisher’s Exact Test.

Among patients who required increased doses of second-generation H1 antihistamines (Phase 2), non-responders had earlier symptom onset, longer disease duration, and a higher prevalence of mental disorders. These factors stood out as possible markers of refractoriness in this therapeutic phase, while the other variables analyzed did not differ statistically (Table 6).

**Table 6:** Comparison of patients who responded versus non-responders to Phase 2 regarding their clinical and laboratory profile (n = 128).

Variable	No (n = 81)	Yes (n = 47)	p	ES
**Sex – n (%):**			0.096^a^	0.147
Female	71 (87.65)	35 (74.47)		
Male	10 (12.35)	12 (25.53)		
**Age in years:**			0.280^b^	0.280^b^
Mean (SD)	44.58 (16.26)	46.01 (19.47)		
Median (Q1; Q 3)	44.00 (38.00; 55.23)	51.00 (34.98; 60.50)		
**BMI (Kg/m^2^):**			0.203^c^	0.235
Mean (SD)	29.17 (6.43)	27.62 (6.84)		
Median (Q1; Q3)	27.60 (24.20; 34.00)	27.90 (23.20; 32.10)		
**Age of onset in years:**			0.036^c^	−0.393
Mean (SD)	30.42 (16.42)	37.26 (19.00)		
Median (Q1; Q3)	30.00 (19.75; 40.00)	42.00 (21.75; 50.00)		
**Time of disease in years:**			0.001^b^	0.285
Mean (SD)	13.38 (10.41)	9.02 (11.34)		
Median (Q1; Q3)	10.50 (5.00; 20.00)	6.00 (2.25; 8.00)		
**Angioedema – n (%)**	66 (81.48)	41 (87.23)	0.549^a^	0.053
**Main diagnoses:**			0.464^a^	0.109
CSU	41 (50.62)	29 (61.70)		
CSU + CIndU	28 (34.57)	12 (25.53)		
CIndU alone	12 (14.81)	6 (12.77)		
**Comorbidities: n (%)**				
Metabolic disorder	40 (49.38)	24 (51.06)	1.000^a^	0.000
Autoimmune disorder	14 (17.28)	13 (27.66)	0.245^a^	0.103
Mental disorder	23 (28.40)#	3 (6.38)#	0.006^a^	0.244
Atopic disorder	28 (34.57)	20 (42.55)	0.478^a^	0.063
Cardiovascular disorder	29 (35.80)	11 (23.40)	0.207^a^	0.111
Hypersensitivity to NSAIDs	25 (30.86)	14 (29.79)	1.000^a^	0.000
**ESR – n (%):**			0.336^a^	0.088
High	35 (46.05)	24 (57.14)		
Normal	41 (53.95)	18 (42.86)		
**CRP – n (%):**			0.075^a^	0.163
Positive	22 (29.33)	6 (13.33)		
Negative	53 (70.67)	39 (86.67)		
**D-Dimer – n (%):**			1.000^d^	0.000
High	8 (25.81)	4 (28.57)		
Normal	23 (74.19)	10 (71.43)		
**Eosinophils:**			0.845^b^	−0.018
Mean (SD)	207.20 (209.86)	210.63 (192.35)		
Median (Q1; Q3):	139.00 (83.50; 273.00)	145.00 (69.92; 276.50)		
**Total IgE:**				
Mean (SD)	246.4 (407.4)	332.9 (579.1);	0.498^b^	−0.068
Median (Q1; Q3)	139.00 (83.50; 273.00)	145.00 (69.92; 276.50)	0.498^b^	−0.068
< 40 – n (%)	15 (29.9)	10 (23.8)	1.000^a^	0.000
≥ 40 – n (%)	43 (74.1)	32 (76.2)	1.000^a^	0.000
**Anti –TPO – n (%):**			1.000^a^	0.000
High	14 (18.67)	8 (20.00)		
Normal	61 (81.33)	32 (80.00)		

SD, Standard Deviation; Q1, First Quartile (25th percentile); Q3, Third Quartile (75th percentile); BMI, Body Mass Index; CSU, Chronic Spontaneous Urticaria; CIndU, Chronic Inducible Urticaria; NSAIDs, Non-Steroidal Anti-Inflammatory Drugs; ESR, Erythrocyte Sedimentation Rate; CRP, C-Reactive Protein; TPO, Peroxidase.

ES, Effect Size. The following effect sizes were calculated: Cohen's d, for the independent *t*-test; r, for the Mann-Whitney test; Cramer's V, for the Fisher's exact test and chi-square test of independence.

a Chi-Square test of independence.

b Mann-Whitney Test.

c Independent *t*-test.

d Fisher’s Exact Test.

For patients who required omalizumab, the presence of mental disorders was also significantly more frequent among patients who did not respond to treatment (Table 7). The other variables also showed no statistical association with treatment response.

**Table 7:** Comparison of patients who responded versus non-responders to Phase 3 regarding their clinical and laboratory profile (n = 58).

Variable	No (n = 11)	Yes (n = 47)	p	ES
**Sex – n (%):**			1.000^a^	0.000
Female	10 (90.91)	44 (93.62)		
Male	1 (9.09)	3 (6.38)		
**Age in years:**			0.056^b^	−0.653
Mean (SD)	35.03 (12.88)	45.37 (16.40)		
Median (Q1; Q3)	39.00 (27.69; 42.50)	45.95 (38.37; 57.61)		
**BMI (kg/m²):**			0.941^b^	−0.025
Mean (DP)	28.72 (7.63)	28.88 (6.28)		
Median (Q1; Q3)	28.90 (22.70; 32.50)	27.20 (24.40; 33.90)		
**Age at onset in years:**			0.494^b^	−0.231
Mean (SD)	25.55 (15.00)	29.15 (15.75)		
Median (Q1; Q3)	25.00 (19.50; 34.50)	30.00 (17.50; 36.75)		
**Time of disease in years:**			0.103^c^	−0.217
Mean (SD)	9.45 (6.44)	15.11 (11.35)		
Median (Q1; Q3):	7.00 (5.00; 16.00)	11.00 (6.00; 21.00)		
**Angioedema – n (%):**	9 (81.82)	41 (87.23)	0.639^a^	0.000
**Main diagnoses**			0.465^a^	0.153
CSU	4 (36.36);	26 (55.32);		
CSU + CIndU	5 (45.45);	16 (34.04);		
CIndU alone	2 (18.18)	5 (10.64)		
**Comorbidities – n (%):**				
Metabolic disorder	5 (45.45)	24 (51.06)	1.000^d^	0.000
Autoimmune disorder	2 (18.18)	7 (14.89)	1.000^a^	0.000
Mental disorder	8 (72.73)#	4 (8.51)#	**<0.001**^a^	0.567
Atopic disorder	4 (36.36)	15 (31.91)	1.000^a^	0.000
Cardiovascular disorder	3 (27.27)	19 (40.43)	0.507^a^	0.061
Hypersensitivity to NSAIDs	3 (27.27)	14 (29.79)	1.000^a^	0.000
**ESR – n (%):**			0.501^a^	0.056
High	3 (30.00)	19 (43.18)		
Normal	7 (70.00)	25 (56.82)		
**CRP – n (%):**			1.000^a^	0.000
Positive	2 (22.22)	13 (28.89)		
Normal	7 (77.78)	32 (71.11)		
**D-Dimer – n (%):**			0.481^a^	0.000
High	1 (50.00)	5 (25.00)		
Normal	1 (50.00)	15 (75.00)		
**Eosinophils:**			0.106^c^	−0.222
Mean (SD)	123.73 (119.26)	230.28 (238.30)		
Median (Q1; Q3)	91.15 (45.25; 130.25)	153.45 (92.88; 280.25)		
**Total IgE:**				
Mean (SD)	229.4 (319.4)	331.6 (506.5)	0.407^c^	−0.134
Median (Q1; Q3)	135.6 (24.0; 280.0)	111.0 (64.8; 419.5)	0.407^c^	−0.134
< 40 – n (%)	3 (37.5)	6 (18.8)	0.348^a^	0.105
≥ 40 – n (%)	5 (62.5)	26 (81.2)	0.348^a^	0.105
**Anti –TPO – n (%):**			0.668^a^	0.039
High	1 (10.00)	9 (20.00)		
Normal	9 (90.00)	36 (80.00)		

SD, Standard Deviation; Q1, First Quartile (25th percentile); Q3, Third Quartile (75th percentile); BMI, Body Mass Index; CSU, Chronic Spontaneous Urticaria; CIndU, Chronic Inducible Urticaria; NSAIDs, Non-Steroidal Anti-Inflammatory Drugs; ESR, Erythrocyte Sedimentation Rate; CRP, C-Reactive Protein; TPO, Peroxidase.

ES, Effect Size. The following effect sizes were calculated: Cohen's d, for the independent *t*-test; r, for the Mann-Whitney test; Cramer's V, for the Fisher's exact test and chi-square test of independence.

a Chi-Square test of independence.

b Mann-Whitney Test.

c Independent *t*-test.

d Fisher’s Exact Test.

Regarding the type of chronic urticaria (CSU, CIndU, or CSU + CIndU), no significant differences were observed in the treatment response profile, whether with standard or increased doses of H1 antihistamines, or with omalizumab (Table 8). Similarly, no statistically significant associations were identified between the omalizumab response subtypes and serum total IgE or anti-TPO levels. Although the different subgroups did not differ overall regarding IgE levels, partial responders had significantly lower mean IgE levels compared to the other response types, with all cases in this group showing levels below 40 IU/mL. No differences were observed between IgE levels in patients with early or late responses, nor between anti-TPO levels in the different response profiles (Table 9).

**Table 8:** Analysis of the association between the pharmacological response profile and the type of CU (n = 175).

Variable	CSU (n = 97)	CSU + CIndU (n = 54)	CIndU (n = 24)	p	V
Responds to high-dose H1 antihistamines	29 (37.18)	12 (30.77)	6 (37.50)	0.927	0.058
Responds to low-dose H1 antihistamines	23 (29.49)	11 (28.21)	5 (31.25)	0.927	0.058
Responds to omalizumab	26 (33.33)	16 (41.03)	5 (31.25)	0.927	0.058

Chi-square test of independence; V, Cramer's V. CSU, Chronic Spontaneous Urticaria; CIndU, Chronic Inducible Urticaria.

**Table 9:** Comparison of serum IgE (n = 40) and Anti-TPO (n = 54) values ​​of patients treated with omalizumab, according to their drug response subtype.

Variable	Early (n = 21)	Late (n = 11)	Partial (n = 2)	Non-responder (n = 6)	p	ES
**Total IgE:**						
Mean (SD)	268.5 (303.1)	452.1 (766.3)	16.8 (17.1)	300.3 (344.5)	0.295^a^	0.020
<40 – n (%)	4 (19.0)	2 (18.2)	2 (100.0)	1 (16.7)	0.122^b^	0.426
≥40 – n (%)	17 (81.0)	9 (81.8)	0 (0.0)	5 (83.33)	0.122	0.426

	Early (n = 33)	Late (n = 13)	Partial (n = 2)	Non-responder (n = 6)	p	V
**Anti-TPO: n (%)**					0.698	0.194
Normal	27 (81.8)	10 (76.9)	2 (100.0)	6 (100.0)		
High	6 (18.2)	3 (23.1)	0 (0.0)	0 (0.0)		

SD, Standard deviation; TPO, Peroxidase.

ES, Effect Size. The following effect sizes were calculated: η2[H], for the Kruskal-Wallis test; Cramer's V, for Fisher's exact test.

a Kruskal-Wallis Test.

b Fisher’s exact Test.

**Fig. 2:**
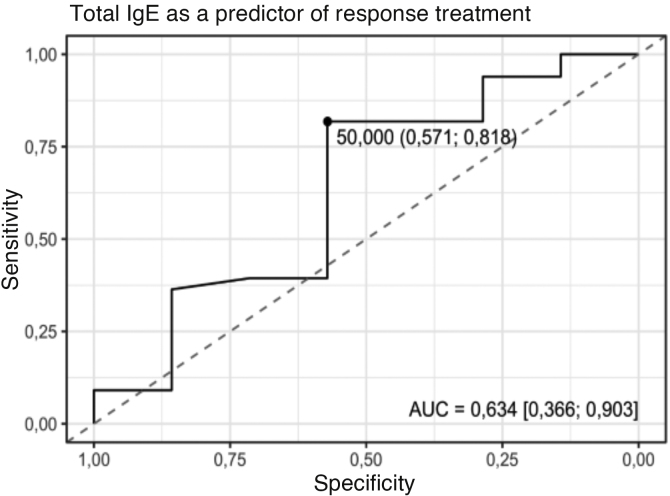
ROC (Receiver Operating Characteristic) curve for Total IgE as a predictor of Response to Treatment. Note: AUC indicates the area under the curve. Values ​​in brackets represent the 95% confidence interval. The highlighted point corresponds to the best cutoff point, according to the Youden index, which simultaneously maximizes sensitivity and specificity. The values in parentheses correspond, respectively, to the specificity and sensitivity for the highlighted cutoff point. The Yes group had, on average, higher Total IgE values than the No group. Source: Prepared by the author.

Finally, the ROC curve analysis evaluating the predictive ability of total IgE in omalizumab response demonstrated limited performance. The area under the curve (AUC) was 0.634 (p = 0.327), with a confidence interval including the 0.5 value, indicating a lack of statistically significant discrimination. The identified cutoff point was 50 IU/mL, with a sensitivity of 81.8% and a specificity of 57.1% (Fig. 2).

## Discussion

This study evaluated clinical and laboratory predictors of treatment response in 175 patients with CU followed at a referral center. The analysis showed that clinical variables such as high BMI, female gender, early symptom onset, prolonged disease duration, and the presence of mental disorders were associated with a worse therapeutic response, especially in the initial phases of treatment with second-generation H1 antihistamines.

Among patients who responded or did not respond to low doses of H1 antihistamines, high BMI was significantly more common in nonresponders, reinforcing previous data associating obesity with greater CU severity, poorer symptom control, and longer disease duration, possibly due to chronic low-grade inflammation with higher concentrations of cytokines such as IL-6.^13^, ^14^

Longer disease duration and early symptom onset were also markers of refractoriness, especially in patients taking increased doses of H1 antihistamines, which is consistent with data from Engstrom et al.,^15^ which indicate worse clinical outcomes in these subgroups. Prolonged duration of chronic urticaria is associated with greater clinical severity, as demonstrated by Toubi et al.,^16^ who observed that moderate to severe forms of CU persisted for significantly longer periods than mild cases. This association may reflect a more intense and sustained inflammatory process, possibly related to autoimmune mechanisms, such as the presence of autoantibodies against the IgE receptor (positive TSA) or antithyroid autoantibodies, both associated with longer disease duration. Additionally, a study showed that young patients with CU (18–40 years) had higher mortality, and that each additional year of disease increases the risk of developing mental disorders by 64% compared to controls.^17^

Furthermore, the higher proportion of women in the group requiring omalizumab may reflect the greater severity of CU in this group, which is also consistent with studies that attribute the higher frequency of refractory forms of the disease to the female sex, possibly due to the higher prevalence of autoimmune diseases among women.^18^

The presence of mental disorders, in turn, was consistently associated with a worse response to both antihistamines and omalizumab. This finding reinforces the importance of neuroimmunoendocrine axes in the pathophysiology of CU and the impact of stress, anxiety, and substance P on mast cell activation.^19^, ^20^ Substance P, in particular, can bind to the MRGPRX2 receptor, present on the surface of mast cells, promoting their exacerbated activation, which may contribute to the persistence of symptoms in these patients.^21^ It is worth noting that the study by Kolkhir et al.^17^ revealed an increased risk of mortality among patients with CSU, including higher rates of suicidal ideation and suicide attempts. This evidence reinforces the need for a multidisciplinary approach in the management of CSU.

Although previous studies^5^, ^6^ have shown that elevated CRP and D-dimer levels, as well as eosinopenia, are associated with poor response to H1 antihistamines, and that low total IgE, elevated CRP/ESR, eosinopenia, and increased D-dimer could predict a lower response to omalizumab and a better response to cyclosporine, none of these associations were confirmed in the present study in the evaluated therapeutic phases. These tests were requested based on the hypothesis that systemic inflammatory processes, reflected by markers such as CRP and ESR,^22^, ^23^ and coagulation activation, evidenced by D-dimer, play a role in the pathophysiology of chronic urticarial.^23^, ^24^ Eosinopenia, in turn, has been associated with increased migration of eosinophils into tissues, which contributes to local inflammation and may indicate increased disease activity, autoimmunity, and poor therapeutic response,^8^, ^25^ while total IgE is considered a possible marker of atopic phenotype and response to omalizumab.^26^, ^27^ However, the present results suggest that, taken alone, these biomarkers have limited predictive value in clinical practice, especially in the present sample. It is important to highlight that the number of patients with D-dimer measurements was small, which may have compromised the analysis. Furthermore, in the present population, eosinophil and total IgE levels may have been higher due to confounding factors, such as the presence of concomitant atopic diseases and intestinal parasitic infections, common in endemic regions.

The analysis of omalizumab response subtypes and IgE levels revealed no statistically significant association, although all partial responders had IgE levels <40 IU/mL. This finding differs from the meta-analysis by Chuang et al.,^28^ but is consistent with studies that also failed to identify this correlation,^9^, ^29^, ^30^ reinforcing the complexity of this relationship. IgE plays a central role in the pathophysiology of CSU, being involved in both autoallergic mechanisms (type I) and type IIb autoimmune forms, where low IgE levels often coexist with IgG autoantibodies against IgE or its receptor (FcεRI).^26^ Therefore, the quantification of total IgE has been proposed as a predictive marker of response to omalizumab, with worse results observed in patients with lower IgE. However, in the present sample, the small number of non-responders and partial responders may have limited the detection of a statistically significant association.

Similarly, no association was observed between anti-TPO levels and response to omalizumab, a result similar to that described by Asero et al.,^31^ but different from that by Kolkhir et al.^32^ The association between thyroid autoimmunity and CU has been described, with several studies demonstrating a high prevalence of IgE and IgG autoantibodies against thyroid antigens (such as TPO and thyroglobulin) in patients with CSU. These autoantibodies can promote mast cell and basophil activation, contributing to the disease pathophysiology.^33^ Although thyroid autoimmunity is associated with greater severity and poorer therapeutic response,^34^ it is unclear whether isolated autoantibodies predict refractoriness. Biomarkers such as BAT, positive TSA, and elevated ANA have been associated with poor response in other studies but were not included in this analysis.

ROC curve analysis for total IgE demonstrated limited performance, with an area under the curve of 0.634 (p = 0.327), confirming its low specificity as an isolated marker of response to omalizumab. Although higher IgE levels are associated with a more favorable response in some studies,^5^, ^35^ these data were not significantly replicated in the present sample, limiting its usefulness as an isolated predictor of response to treatment with this drug.

Therefore, the results reinforce the hypothesis that clinical and psychosocial characteristics are, to date, the main predictors of therapeutic response in chronic urticaria. The cross-sectional design, the small number of non-responders to omalizumab, and the lack of data on the duration of drug exposure are limitations that should be considered. In addition to these limitations, it is important to recognize that the study was conducted with a convenience sample recruited at a specialized tertiary center, which may have generated selection bias, limiting the representativeness of the general population with chronic urticaria. To minimize this bias, well-defined inclusion and exclusion criteria were used, with data collection performed in a standardized manner by a trained team using validated instruments (UCT and AECT). The potential information bias is also recognized, inherent to the use of medical record data, which was mitigated by systematically reviewing records and supplementing information during medical consultations, always with the patients’ prior consent.

Regarding external validity, because this is a single-center study, extrapolation of the results to other populations should be done with caution, taking into account the specific clinical and epidemiological profile of the study sample. On the other hand, internal validity was ensured by adopting diagnostic criteria based on international guidelines (EAACI/GA²LEN/EDF/WAO), using standardized therapeutic protocols, and by applying validated instruments to assess treatment response.

Prospective studies with standardized biomarker assessment are required to validate the findings and guide precision medicine in CU management.

## Conclusion

The study aimed to analyze predictive markers of treatment response in patients with chronic urticaria. The research reinforces the complexity of CU and its management challenges, highlighting its predominance in women, adult onset, and long disease duration. The main predictors of poorer response to treatment were longer disease duration, female sex, high BMI, early symptom onset, and the presence of mental disorders. Eosinophils, total IgE, and the other evaluated laboratory markers (ESR, CRP, D-dimer, and anti-TPO) showed no significant association with therapeutic response.

## ORCID IDs

Joanemile Pacheco de Figueiredo: 0000-0002-1899-8935

Leila Vieira Borges Trancoso Neves: 0000-0003-2364-2391

José Carlisson Santos de Oliveira: 0000-0003-4040-8257

Janinne Souza de Oliveira: 0009-0002-0108-945X

Vitória Rani Figueiredo: 0009-0002-9518-2806

Régis de Albuquerque Campos: 0000-0001-9524-761X

## Authors' contributions

Joice Trigo da Fonseca: Design and planning of the study; drafting and editing of the manuscript; collection, analysis, and interpretation of data; intellectual participation in the propaedeutic and/or therapeutic conduct of the studied cases; critical review of the literature; critical review of the manuscript; statistical analysis; approval of the final version of the manuscript.

Joanemile Pacheco de Figueiredo: Collection, analysis, and interpretation of data; intellectual participation in the propaedeutic and/or therapeutic conduct of the studied cases; critical review of the manuscript; approval of the final version of the manuscript.

Leila Vieira Borges Trancoso Neves: Collection, analysis, and interpretation of data; intellectual participation in the propaedeutic and/or therapeutic conduct of the studied cases; critical review of the manuscript; approval of the final version of the manuscript.

José Carlisson Santos de Oliveira: Collection, analysis, and interpretation of data; intellectual participation in the propaedeutic and/or therapeutic conduct of the studied cases; critical review of the manuscript; approval of the final version of the manuscript.

Janinne de Souza Oliveira: Collection, analysis, and interpretation of data; critical review of the literature. Critical review of the manuscript; approval of the final version of the manuscript.

Vitória Rani Figueiredo: collection, analysis, and interpretation of data; critical review of the literature; critical review of the manuscript; approval of the final version of the manuscript.

Régis de Albuquerque Campos: Design and planning of the study; effective participation in research orientation; collection, analysis, and interpretation of data; intellectual participation in the propaedeutic and/or therapeutic conduct of the studied cases; critical review of the literature; critical review of the manuscript; approval of the final version of the manuscript.

## Financial support

None declared.

## Research data availability

The entire dataset supporting the results of this study was published in this article.

## Conflicts of interest

Campos RA has declared receiving speaker fees from Novartis, Takeda, and Sanofi.

Oliveira JCS has declared receiving speaker fees from Takeda, Sanofi, Novartis, and AbbVie.

The remaining authors declare that the research was conducted in the absence of any commercial or financial relationships that could be construed as potential conflicts of interest.
